# Fulminant Mycosis Fungoides with Tissue Eosinophilia: A Unique Presentation of Two Cases with Acro-Periorbital Ulceration and An Aggressive Clinical Course

**DOI:** 10.4172/2155-9929.1000337

**Published:** 2017-03-02

**Authors:** David R Pearson, Mayumi Fujita, Whitney A High

**Affiliations:** University of Colorado School of Medicine in Aurora, USA

**Keywords:** Eosinophilia, Mycosis fungoides, Clinicopathologic correlation

## Abstract

We describe two unique cases of fulminant mycosis fungoides with remarkably similar and aggressive clinical courses resulting in death. Both cases demonstrated ulcerated palmar and periorbital plaques and marked tissue eosinophilia, which was confirmed by T-cell receptor γ chain gene rearrangement studies to display identical monoclonality at temporally and anatomically distinct sites. Dense eosinophilic infiltrates on biopsy led to misdiagnosis of inflammatory dermatoses in both instances.

While mycosis fungoides may be challenging to diagnose histologically, the presence of eosinophils in progressive disease may herald a poor prognosis and should not exclude the diagnosis.

## Introduction

Mycosis fungoides (MF) and its most common variants, folliculotropic mycosis fungoides, pagetoid reticulosis, and granulomatous slack skin, compose slightly less than half of cutaneous lymphomas [1]. Pimpinelli et al. summarized the 2005 International Society for Cutaneous Lymphoma (ISCL) consensus algorithm for diagnosis of MF, taking into account clinical, histologic, immunohistochemical, and genetic findings [2]. Despite these guidelines, MF can be challenging to diagnose and may mimic inflammatory dermatoses both clinically and histologically, including eczematous or lichenoid dermatoses, psoriasis, dermatophytoses, lymphomatoid papulosis, B-cell lymphoma, and others [3–8]. Further challenges arise as MF commonly loses prototypical T-cell surface antigens such as CD7 [9–13].

Tissue eosinophilia can be a useful biomarker in distinguishing MF from inflammatory dermatoses, and among other metrics, the presence of eosinophils has been used as a minor criteria for excluding MF [14,15]. However, studies suggest that as MF progresses from early (patch and plaque) to advanced (tumor, erythroderma, and Sezary syndrome) stage disease, tissue eosinophils become more prominent [15–18].

Here we describe two cases of plaque stage MF with marked tissue eosinophilia that followed an impressively similar and aggressive clinical course. Both patients were initially misdiagnosed with inflammatory dermatoses due to their atypical histologic presentations. A high index of clinical suspicion in combination with T-cell receptor (TCR) γ chain gene rearrangement studies and progressive disease eventually led to the correct diagnosis.

## Case Series

### Case 1

**Figure 1:**
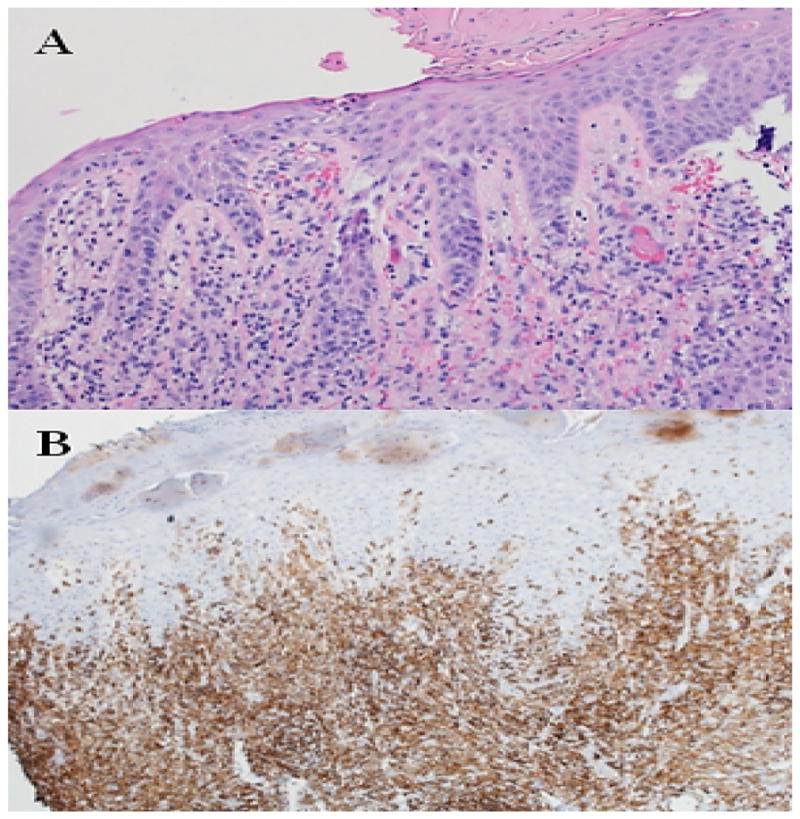
Biopsy results of case 1 during hospitalization. A) A dermal infiltration by large pleomorphic cells overlying a mixed inflammatory infiltrate with eosinophils. B) Numerous CD4 positive cells are present in the dermis with fewer scattered in the epidermis.

A 72-year-old man with a remote history of melanoma was admitted for malodorous ulcerations on the hands. Wound cultures upon admission grew methicillin-resistant *Staphylococcus aureus* and multidrug-resistant *Pseudomonas aeruginosa*. The patient was placed upon intravenous antibiotics. Laboratory testing revealed leukocytosis (16,500/μL) with profound peripheral eosinophilia (4600/μL). Lactate dehydrogenase (LDH) was within normal limits. On exam the patient was nearly erythrodermic with ulcerated palmar plaques. Biopsy showed dermal infiltration by numerous large atypical cells overlying a mixed inflammatory infiltrate with eosinophils (Figure 1). The atypical cells were CD4 positive, CD3, CD7, CD8, CD20, and S100 negative. There were scattered cells that stained with CD30 and CD68. Consultation from a reference lab was obtained, and a presumptive diagnosis of non-Langerhans cell histiocytosis was rendered. Upon chart review, the ulcerations had first been noted on his palms 2 years before admission by his medical oncologist. By report, that biopsy sample demonstrated a mixed chronic inflammatory infiltrate with CD3, CD4, and CD7 positive lymphocytes, numerous CD68 positive histiocytes, and abundant eosinophils.

**Figure 2:**
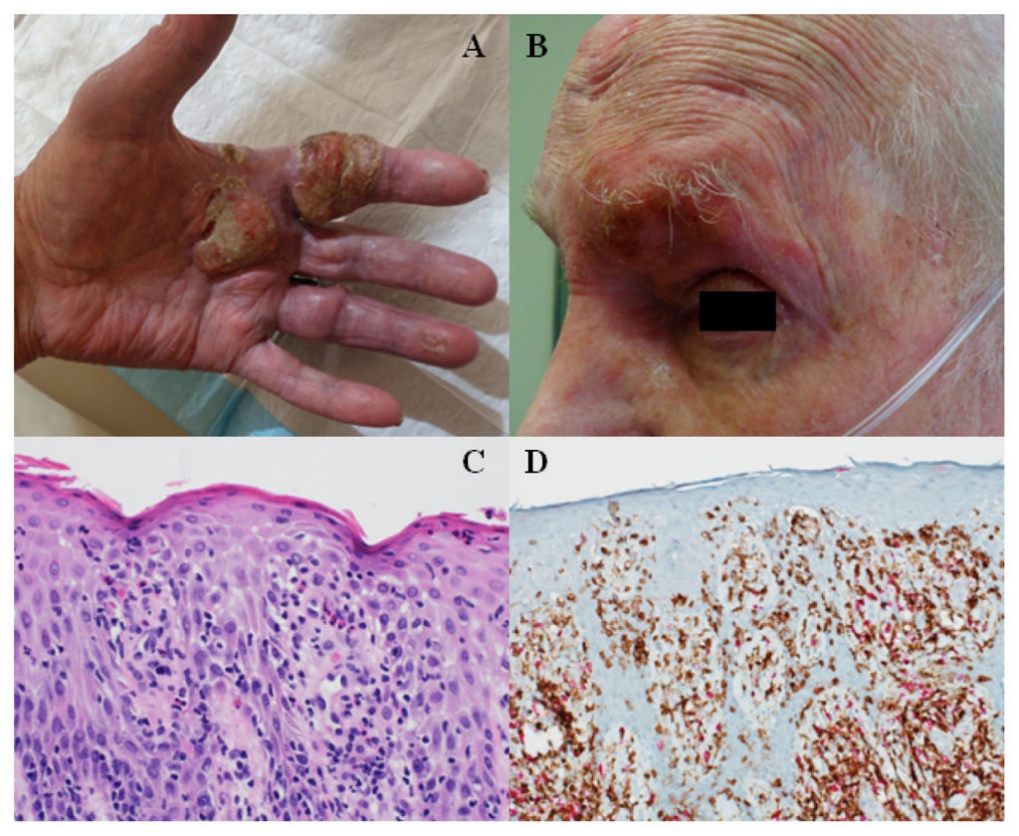
Case 1 in dermatology clinic following hospitalization. A) Large ulcerated plaques on the palmar surface, with B) periorbital erythema and crusting. C) Biopsy results demonstrate an atypical lymphoid infiltrate with hyperchromasia, pleomorphism, and epidermotropism. Numerous eosinophils are noted. D) Immunohistochemistry highlights epidermotropism of CD4 positive cells (brown) and associated CD8 positive cells (pink).

After stabilization and transition to oral antibiotics, the patient was discharged. He followed up in clinic, where enlargement of palmar lesions and new ulcerating plaques on the upper extremity and periorbital area were noted (Figure 2A and 2B). Due to clinicopathologic dissonance, repeat biopsies were performed on the palmar plaques as well as scattered erythematous patches on the trunk. These biopsies demonstrated an atypical lymphoid infiltrate with epidermotropism and numerous eosinophils (Figure 2C and 2D). TCR γ chain gene rearrangement studies were performed on specimens from his initial biopsies 2 years prior, those obtained during his hospitalization, and clinic biopsies from both the palmar plaques and erythematous patches on the trunk. All studies demonstrated identical monoclonality based on amplicon size. The patient was diagnosed with MF. PET-CT revealed two FDG-avid nodules in the lower extremities suspicious for lymphoma. He was initiated on romedepsin chemotherapy, with 22 mg delivered intravenously once weekly for 3 weeks followed by 1 week off. During his second and third treatment cycles, his course was complicated by recurrent superficial skin infections and a relentlessly progressive deterioration in his health and function. He was placed on comfort care and expired shortly thereafter.

### Case 2

**Figure 3:**
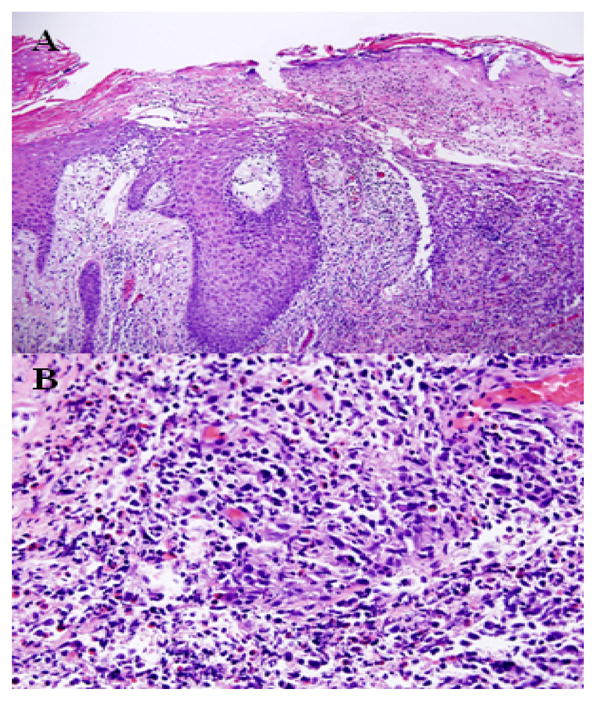
Biopsy results of case 2 upon initial presentation. A) At medium power, there is ulceration of the epidermis with spongiosis and a mixed inflammatory infiltrate. B) Higher power demonstrates numerous eosinophils and mild lymphocyte pleomorphism.

A 65-year-old man presented to clinic for evaluation of a small non-healing ulcer on the right palm which had been present for 8 weeks. A shave biopsy demonstrated superficial ulceration, copious dermal eosinophils, and eosinophilic cytoplasmic inclusions within keratinocytes (Figure 3). Due to his rural lifestyle, there was high suspicion of parapoxvirus infection overlying presumed hand eczema.

Six weeks later the patient returned to clinic with worsening of his palmar lesion and was noted to have bilateral epitrochlear lymphadenopathy. A second biopsy obtained from the enlarged palmar lesion also demonstrated a dense infiltration of eosinophils with a modest number of large atypical lymphocytes. Immunohistochemical staining revealed an admixture of CD3 positive cells and CD20 positive cells, and negative staining with CD30. TCR γ chain gene rearrangement studies from the palmar biopsy demonstrated monoclonality. The earlier shave biopsy was reviewed retrospectively and the same atypical lymphocytes were appreciated. Full body CT scan revealed bilateral axillary and inguinal adenopathy, and a PET scan showed increased metabolic activity in the right occipital region and several foci in all four extremities.

**Figure 4:**
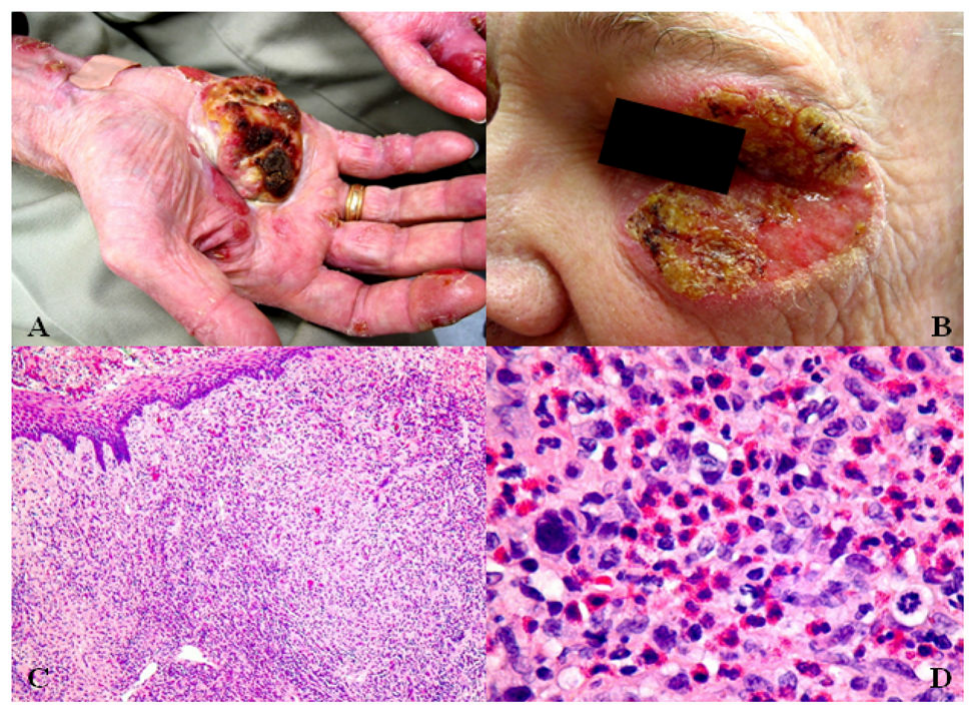
Case 2 at his second follow-up visit. A) Large ulcerated palmar plaques, with B) marked periorbital involvement. C) Biopsy specimens demonstrate a mixed inflammatory infiltrate and numerous eosinophils at low power. D) High power reveals a dense eosinophilic infiltrate and many atypical lymphocytes with hyperchromasia, pleomorphism, and abnormal mitotic figures.

Four weeks later, he was again evaluated in dermatology clinic and was found to have progressed (Figure 4A and 4B). Incisional biopsy of the right hand demonstrated a dense inflammatory infiltrate in the dermis with a prevalence of eosinophils and medium to large-sized lymphocytes with nuclear pleomorphism and frequent mitoses (Figure 4C and 4D). These abnormal lymphocytes were positive for CD4, but negative for CD5, CD8, CD30, CD56, and EBER *in situ* hybridization. TCR γ chain gene rearrangement studies from the right hand displayed identical monoclonality to the prior biopsy specimens based on amplicon size.

The patient elected to pursue alternative medicine options and he was lost to follow-up for 4 months. When he returned to clinic, his lesions had progressed. Comfort care was recommended and the patient expired.

## Discussion

Both cases demonstrate an unusual variant of aggressive plaque stage MF with a heavy infiltration of tissue eosinophils, manifesting with acrally-distributed ulcerative plaques, notable periorbital involvement, and eventual nodal metastases. Due to tissue eosinophilia, both patients were initially misdiagnosed with inflammatory dermatoses. High clinical suspicion for cutaneous lymphoma with multiple biopsies and demonstration of T-cell monoclonality from anatomically and temporally distinct sites confirmed the diagnosis of MF in both cases and was critical to the final assessment.

The incidence of monoclonality in TCR γ chain gene rearrangement in MF varies by clinical stage, with 50% of patch stage, 73% of plaque stage, and 83% to 100% of tumor stage demonstrating monoclonality [13]. TCR studies are particularly valuable in establishing the correct diagnosis but must be interpreted in the context of clinical, histologic, and immunohistochemical data, because T-cell monoclonality can also be found in 25% to 65% of various inflammatory dermatoses [19–22]. Establishing identical monoclonality from two anatomically distant sites, however, increases sensitivity and specificity for MF to 82.6% and 95.7%, respectively [23].

Tissue eosinophils may become numerous with progression of MF to advanced stage tumors and folliculotropic variants [1], but are uncommon in early stage disease [15–18]. Longstanding MF may progress to a predominantly Th2 immunophenotype, with interleukin (IL) 4 secreting T-cells, eosinophilia, erythroderma, and increased susceptibility to infection [16,24–26]. Blood and tissue eosinophilia at diagnosis has been implicated as a poor prognostic indicator [27–30].

In our cases, disease progression occurred relatively rapidly in both patients within several months, resulting in patient death. To the authors’ knowledge, there have not been prior reported cases of fulminant mycosis fungoides with this clinical pattern of acrally-distributed ulcerative plaques, prominent periorbital involvement, and rapid progression to death. When tissue eosinophilia is observed with this clinical presentation, a poor prognosis may be expected.

MF may be challenging to diagnose without contributing evidence from clinical, histologic, immunohistochemical, and genetic datasets. It is important to underscore that these metrics must not be obtained in isolation but instead interpreted in context. Since MF may masquerade as inflammatory or other neoplastic dermatoses, multiple series of biopsies are often required to correctly establish the diagnosis. Our cases highlight that when faced with clinicopathologic dissonance, astute clinicians must rely on the composite of data to form the correct diagnosis. In particular, the presence of tissue eosinophilia should not be used as a criterion for exclusion of MF in an appropriate clinical scenario.

## Abbreviations

CD: Cluster of Differentiation
EBER: Epstein-Barr virus Encoded RNA
FDG: Fludeoxyglucose
MF: Mycosis Fungoides
PET-CT: Positron Emission Tomography Computed Tomography
TCR: T-Cell Receptor
